# Heatwave‐Induced Paternal Effects Have Limited Adaptive Benefits in Offspring

**DOI:** 10.1002/ece3.70399

**Published:** 2024-10-20

**Authors:** Sara D. Irish, Andreas Sutter, Livia Pinzoni, Mabel C. Sydney, Laura Travers, David Murray, Jean‐Charles de Coriolis, Simone Immler

**Affiliations:** ^1^ School of Biological Sciences University of East Anglia Norwich UK

**Keywords:** adaptive response, embryo viability, global warming, male fertility, paternal effects, thermal fertility limit

## Abstract

As the threat of climate change and associated heatwaves grows, we need to understand how natural populations will respond. Inter‐generational non‐genetic inheritance may play a key role in rapid adaptation, but whether such mechanisms are truly adaptive and sufficient to protect wild populations is unclear. The contribution of paternal effects in particular is not fully understood, even though the male reproductive system may be highly sensitive to heatwaves. We used the zebrafish *Danio rerio* to investigate the effects of heatwaves on male fertility and assess potential adaptive benefits to their offspring in a number of large‐scale heatwave experiments. Heatwave conditions had negative effects on male fertility by reducing gamete quality and fertilisation success, and we found indications of an adaptive effect on hatching in offspring produced by heatwave‐exposed males. Our findings highlight the importance of including male and female fertility when determining species ability to cope with extreme conditions and suggest that parental effects provide limited adaptive benefits.

## Graphical abstract

As the threat of climate change advances, understanding how extreme weather events influence wild populations is integral. Our study shows that heatwaves negatively influence not only male fertility in a freshwater fish, but also affect the offspring of heatwave‐exposed parents. While heatwave‐exposed males may confer limited adaptive benefits to their offspring, stronger detrimental maternal effects following heatwaves may override those benefits.

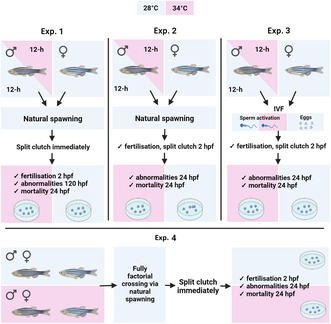

## 1 Introduction

Wild animal populations are facing growing challenges due to climate change (IPCC 2023) and extreme weather events are becoming more frequent, intense and widespread (Frich et al. 2002). Populations typically respond to climatic challenges through range shifts, or experience declines and potentially extinction (Parmesan and Yohe 2003). However, range shifts alone may not provide sufficient protection to populations against the rapid onset of extreme weather events and may become more difficult as habitats shrink due to anthropogenic causes (IPCC 2023). Therefore, many populations will need to adapt to avoid declines and extinction risks. It remains unclear whether adaptation can happen quickly enough to keep pace with climate change effects (Bridle and Vines 2007), since extreme weather events, such as heatwaves, may be too random or rare to evoke appropriate responses (Van De Pol et al. 2017). Furthermore, the high sensitivity of the reproductive system to temperature stress makes it unclear whether such adaptations can spread and persist in populations.

Recently, it has been implicated that fertility plays a crucial role in the survival of populations under thermal stress (Walsh et al. 2019). Traditionally, temperature ranges suitable for populations have been derived using Critical Thermal Limits (CTLs), the temperature range within which an organism can function physiologically to survive (Kellermann et al. 2012; Bennett et al. 2021). The increased somatic maintenance costs expected to occur under thermal stress, however, could reduce fertility via diversion of resource allocation (Kirkwood 1977) and hence impede population growth. Walsh et al. (2019) proposed that Thermal Fertility Limits (TFLs), the temperature range within which an organism can reproduce, may be a better estimate of temperature niches, as fertility will likely be affected at a lower temperature threshold than survival (Walsh et al. 2019). Indeed, across *Drosophila* species, TFLs of male flies better predicted their current species ranges with at least 35% greater accuracy than CTLs (Parratt et al. 2021). This link between fertility and thermal stress indicates that populations could be more sensitive to temperature effects than previously thought.

The effects of environmental change on male fertility have garnered attention over recent years due to the expected heightened sensitivity of the male reproductive system compared to that of females, which may be comparatively resilient (Iossa 2019; Walsh et al. 2019). Sperm morphology and motility are key determinants of fertility and are vulnerable to external stressors in many taxa (Reinhardt, Dobler, and Abbott 2015). Heatwaves in particular were shown to reduce sperm production, competitiveness and overall male fertility in the ectotherm red flour beetle *Tribolium castaneum* (Sales et al. 2018) and the Mediterranean field cricket *Gryllus bimaculatus* (Gasparini et al. 2018). On the other hand, males who continually produce new sperm may have a greater potential for recovery from environmental effects compared to females (Sales, Vasudeva, and Gage 2021).

Temperature‐related stress on the reproductive system may not only reduce fertility, but also influence offspring fitness (Hu and Barrett 2017; Lande and Kirkpatrick 1990; Porcelli et al. 2015). Heritable factors that alter gene regulation, but not the genetic code, can allow for the transmission of environmentally induced traits across generations (Avital and Jablonka 2000; Bonduriansky and Day 2009). In fact, in Brook char *Salvelinus fontinalis*, parents matured at high temperatures produced offspring with 188 differentially methylated regions compared to offspring produced by parents matured in cold temperatures (Venney et al. 2022), indicating that temperature can induce heritable non‐genetic effects. Often, these parental effects are referred to as “anticipatory” and have been demonstrated in several species (Marshall and Uller 2007). However, in order for anticipatory effects to be beneficial, the environmental conditions of parents and offspring must match (Gasparini et al. 2018; Silva, Otto, and Immler 2021). Thus, species found in regions, such as the tropics, where they are less likely to endure extreme seasonal fluctuations, may more easily be able to anticipate the environment of their offspring. For example, coral reef damselfish *Acanthochromic polyacanthus* exhibited higher reproductive success at higher temperatures when their grandparents and parents were also kept at higher temperatures (Munday, Donelson, and Domingos 2017). However, other evidence suggests that tropical fish, like *Danio rerio*, may have hard limits to how adaptation could protect populations, as after several generations of selection for increased upper thermal tolerance their capacity to acclimate declined (Morgan et al. 2020). Species that do typically experience extreme seasonal fluctuations in their environments may already carry an increased capacity for plasticity. In the sheepshead minnow *Cyprinodon variegatus*, known for its eurythermal nature (Bennett and Beitinger 1997), offspring reared in temperatures that match that of their parents had the fastest growth rates, which is highly correlated with fitness in this species (Salinas and Munch 2012). The adaptive potential of inherited factors resulting from plastic environmental responses is highly dependent on not only the speed and pattern of environmental change, but also the environment that organisms are already adapted to.

In addition to environmental differences, unique physiologies between species and sexes also likely impact the inheritance of parental effects. It is widely accepted that mothers can transmit effects via biomolecules such as nutrients and hormones or maternal care to their gametes and thus offspring. Paternal transmission is more controversial (Crean, Dwyer, and Marshall 2013) and has long been regarded as a rare phenomenon confined to species exhibiting paternal care (Crean and Bonduriansky 2014; Marshall 2015). However, sperm contain a complex range of biomolecules that can influence offspring development both directly through phenotypic changes (Avila et al. 2011; Immler 2018; Michaud et al. 2013) and indirectly via female‐dependent effects where female fitness or behaviour is altered (Perry, Sirot, and Wigby 2013). Sperm also carry epigenetic information such as small RNAs or methyl groups (Godden and Immler 2023; Immler 2018) that may be influenced by environmental stressors. In the Mediterranean mussel *Mytilus galloprovincialis*, post‐ejaculation thermal stress resulted in sperm with reduced *hsp90* mRNA, which is indicative of either a stress response or mRNA degradation (Lymbery, Evans, and Kennington 2020). While this did not have any phenotypic effects on the sperm themselves, there could be an effect on offspring phenotype (Bonduriansky and Day 2009; Curley, Mashoodh, and Champagne 2011; Rando 2012).

Some studies indicate that environmental temperature fluctuations may be enough to mediate paternally induced phenotypic effects in offspring, but the role of these effects varies. In the Mediterranean mussel for example, males exposed to thermal stress produced higher‐quality offspring following sperm thermal stress, but this may be due to selective disappearance rather than inherited adaptive phenotypes (Lymbery, Kennington, and Evans 2021). However, offspring sired by heat‐treated sperm did not perform better than control offspring in the face of thermal stress. In the field cricket, offspring of heat‐treated males were observed to have lower survival and hatching success (Gasparini et al. 2018) and in the marine tubeworm *Galeolaraia caespitosa*, acute paternal thermal stress reduced offspring survival, but paternal acclimation to higher temperatures mitigated this effect (Guillaume, Monro, and Marshall 2016). Beyond developmental effects, late‐life effects were observed in the offspring of heat‐treated male flour beetles, which exhibited reduced reproductive capacity and lifespan (Sales et al. 2018). While in recent years the body of evidence for paternal effects has grown, evidence of their adaptive role is still unclear (Immler 2018).

External fertilisers represent ideal study systems to investigate the environmental effects on the fertility of both sexes, as gametes of these species experience direct environmental exposure, making them particularly vulnerable to environmental conditions (Crean and Immler 2021; Walsh et al. 2019). In this study, we exposed male and female zebrafish *Danio rerio* to simulated heatwaves to investigate the effects of periods of high temperature on male and female fertility and offspring performance and resilience. Zebrafish are an aquatic model system for environmental effects that naturally experience heatwaves in their natural habitats across India and Pakistan (Morgan et al. 2019). We ran four separate experiments exposing adult fish, their sperm and offspring to standard (28°C) and high (34°C) temperatures to identify the stages that are the most vulnerable to temperature changes from adult fish to gametes and early offspring, and to disentangle the relative importance of paternal and maternal effects for offspring performance. If parental effects are adaptive, offspring should perform better than control offspring in temperature environments that match that of their parents. Alternatively, if parental effects are maladaptive, their offspring should perform worse than controls regardless of their temperature environment.

## 2 Material and Methods

### 2.1 Animal Husbandry

We performed four major experiments where we used adult (6–18 months old) and embryo/larvae (0–5 days post‐fertilisation) zebrafish of the AB wildtype strain. The fish were originally obtained from the European Zebrafish Resource Center (EZRC) in Karlsruhe, Germany and carefully bred following a pedigree to avoid inbreeding for up to two generations at the Constant Environment Facilities at the School of Biological Sciences, University of East Anglia. Prior to the experiment, fish were kept in 50:50 male–female ratios in 3 and 10‐L tanks in the zebrafish rack system (Tecniplast) equipped with an automated filtering cycle and constant water flow at a temperature of 28°C–28.5°C and a 14:10 dark–light cycle. The fish were fed three to four times per day on a mixed diet consisting of high‐protein dried food (Sparos) and live brine shrimp (*Artemia*).

### 2.2 Overview of Experiments

We performed four separate experiments that differ in aim and focus:

*Experiment 1 (Exp. 1)*: we focused on male effects and tested whether paternal heatwave exposure would result in increased offspring survival in warmer environments using natural spawning and the effect of elevated temperature on sperm phenotypes and sperm genome integrity.

*Experiment 2 (Exp. 2)*: we repeated offspring assays from Exp. 1 to confirm the results.

*Experiment 3 (Exp. 3)*: we used IVF following heatwave exposure to rule out potential confounding effects of paternal behaviour. We also added a temperature manipulation step during sperm activation for IVF to investigate the potential for environmentally induced heritable effects at the sperm level.

*Experiment 4 (Exp. 4)*: we used both male and female fish exposed to heatwaves to test for the relative importance of maternal and paternal effects on offspring resilience to heatwaves during early development via a fully factorial crossing of parents across treatments.

### 2.3 Experimental Procedure

**FIGURE 1:**
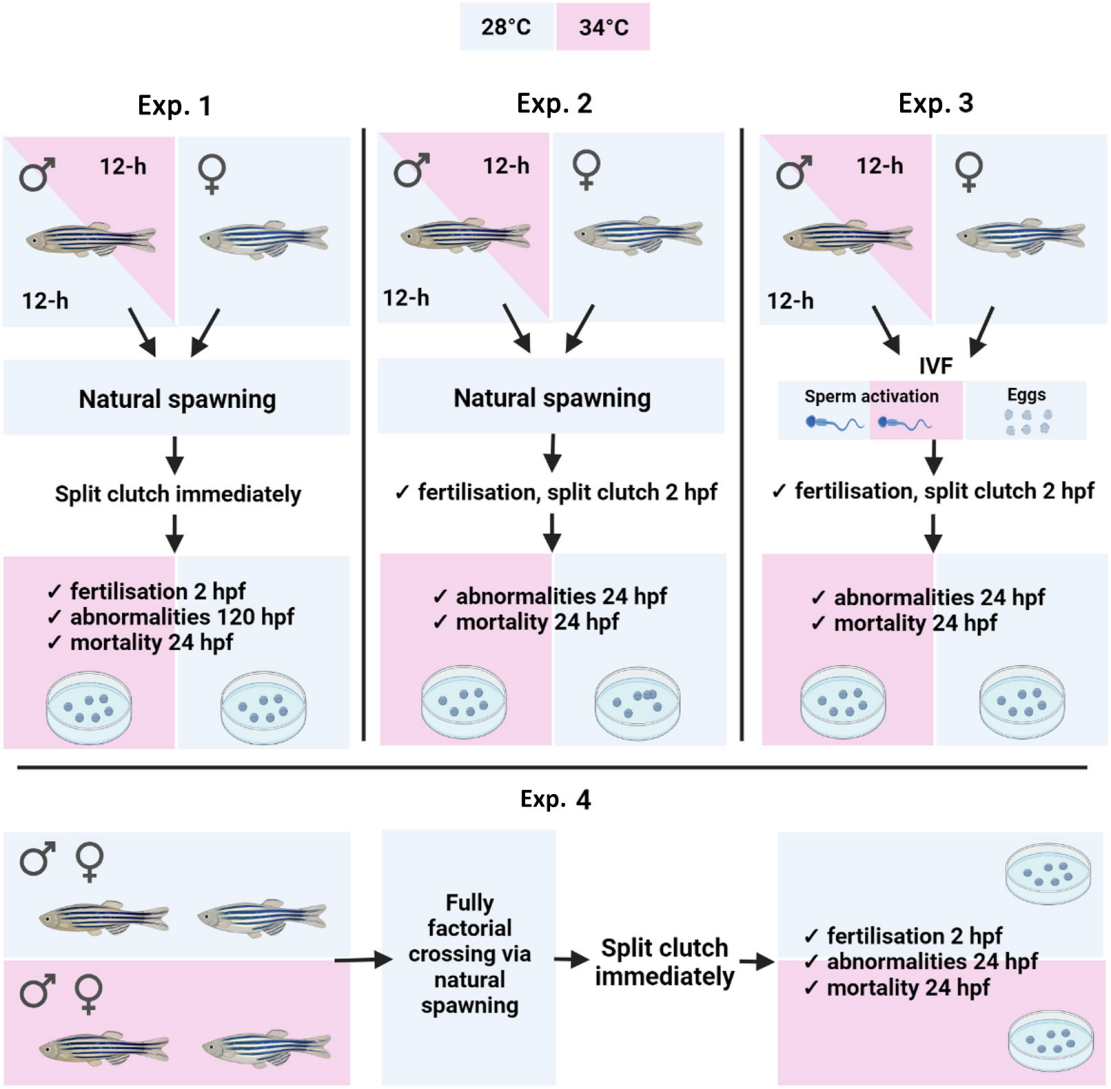
Experimental design for Experiments 1–4. Blue represents the control (28°C) temperature and pink represents the high‐temperature (34°C) treatment. Unless otherwise denoted, the colour represents a constant temperature. Image created with BioRender.com.

For all experiments, we kept groups of adult fish in their given treatments for 2 weeks (Figure 1). The duration of the spermatogenic cycle in zebrafish is approximately 6 days (Leal et al. 2009), and this two‐week period ensured at least one full spermatogenic cycle occurred during this treatment time. Control fish were maintained at 28°C, and water temperatures of 34°C were used as our heatwave treatments. Both temperatures are within the natural range encountered by zebrafish in their native environment (Morgan et al. 2019). In Exp. 1, 2 and 3, adult zebrafish were exposed to diurnal heatwaves. To simulate heatwaves (Monday to Friday only), we switched on 50‐W Aqua‐nano heaters (Aquadistri UK Ltd) within the tanks shortly after artificial dawn, which gradually increased the temperature to around 34°C (point measurements 33.8 ± 0.6°C mean ± SD) over a period of around an hour. It has rather mild effects on embryo viability but pronounced negative effects on development (Pype et al. 2015). Ten hours later, heaters were switched off, reducing the temperature in the tanks back to the system temperature of 28°C–28.5°C. The control treatment included introduced heating elements that were not switched on (temperature mean ± SD = 28.2 ± 0.2°C). To maintain the dissolved oxygen concentration at similar levels in control and high‐temperature treatments, we used air pumps in all tanks. Heatwave and control groups were maintained under the respective regimes for 2 weeks. In the heatwave treatment for Exp. 4, fish of both sexes were exposed to high temperatures (34°C) throughout the full duration of 2 weeks without diurnal variation to strengthen the effects, allowing us to disentangle maternal and paternal effects in the resulting offspring.

### 2.4 Reproduction Assays

At the end of the two‐week period, adult fish were set up for reproduction, either by natural spawning or by IVF as follows (see Figure 1 and Table A1 in Appendix for overview):

*Natural spawning*: In Exp. 1, 2 and 4, one male and one female were kept overnight in a breeding tank maintained at ~28°C with a transparent separator between them. The separator was removed at artificial dawn the following morning. Eggs were collected within 1 h after spawning using a fine‐meshed sieve. Eggs were then treated with an antifungal agent (methylene blue) and split between two Petri dishes for incubation at control (28°C) and treatment (34°C) temperatures. In Exp. 1 and 4, clutches were placed into control and heatwave temperatures upon retrieval and splitting, whereas in Exp. 2, they were all incubated at 28°C until 2 hpf and then split into the two treatments. We specifically tested whether this initial difference would have a downstream effect, but we found no evidence (χ^2^_1_ = 1.554, *p* = 0.213).

*IVF*: In Exp. 3, we followed procedures as described in (Alavioon et al. 2017) with slight modifications. The evening before IVF, we separated males into same‐sex groups of three individuals. Stock females that displayed a bulging abdomen were set up individually in small tanks, separated with a transparent divider from a companion male who provided visual and chemical stimuli. As oviposition is induced by morning light, tanks for containing fish for IVF were kept under a black cover until use.

For gamete collection, fish were anaesthetised in a 1.0–3.0 mg/L metomidate hydrochloride (AquacalmTM) solution until gill movement stopped, but for no longer than 2 min. Anaesthetised males were washed in fresh tank water and placed on a soft and wet sponge with their anal fin and cloaca exposed under a dissecting microscope (Nikon SMZ800). The anal fin and lower stomach were dried using paper towels to prevent sperm activation upon contact with water. The males were squeezed gently in craniocaudal direction and the ejaculation was collected into a small capillary (Sigma‐Aldric, P0674). The ejaculate was then transferred into a 0.2 mL Eppendorf tube containing 20 μL of Hank's buffer (HBSS) and kept on ice until use within 1–2 h of collection. We collected as much ejaculate as possible from each male, taking note of the estimated quantity using a visual scoring system (0–4 mm with 0.5 mm increments).

For egg collection, females were gently dried around the belly and the papilla with paper towel and positioned on their side inside a large plastic weighing boat. Using a wet finger, we gently squeezed the females' abdomen to release eggs. Upon release, eggs were transferred with the tip of a wet paintbrush into two small wells in a wax plate to split the clutch into two halves for IVF.

We performed IVF within a minute of eggs being collected, carrying out procedures (apart from sperm activation) on a warming plate set to 28.5°C. Two experimenters worked simultaneously to synchronise the sperm activation treatments. Two 20 μL semen‐buffer samples were activated in 60 μL of fish water that had been kept in 0.5 mL Eppendorf tubes in water baths set to 28°C and 34°C, respectively. After about 10 s, each activated semen‐water subsample was added to one of the two wells containing half of the eggs, alongside 120 μL control temperature fish water, to minimise the temperature difference between the eggs of either sub‐clutch. We randomised the treatments between experimenters and the wells between treatments. After 90 s, each sub‐clutch was further split into two halves, allowing a two‐by‐two‐by‐two fully factorial experimental design with two male, two sperm and two embryo‐rearing temperature treatments.

### 2.5 Assays to Measure Performance in Embryos and Larvae

Clutches obtained from natural spawning and IVF were split into two halves to expose half of the offspring to 28°C and the other half to 34°C. We checked eggs for fertilisation rate at 2 hpf, and for survival and normal development at 24 hpf (Exp. 2, 3 & 4) and normal development at 120 hpf (Exp. 1). Developmental abnormalities were determined as any deviations from Kimmel et al.'s (1995) description of the stage of embryonic development. Embryos were incubated at control (28°C) or elevated (34°C) temperatures, starting directly after egg collection (Exp. 1 & 4) or 2 hpf (Exp. 2 & 3). Dead and unfertilised eggs were removed during checks. Dead eggs generally could not be definitively identified as abnormal, and thus were not included in abnormal offspring counts. Additionally, in Exp. 4, incidences of early hatching were recorded at 48 hpf.

Data collection was double‐blinded in experiments wherever possible. Experimenters were blind with respect to adult treatment during IVF, egg collection and embryo scoring, and blind with respect to sperm activation temperature during embryo scoring.

### 2.6 Sperm Motility

Sperm samples for motility and velocity analyses were collected as described above for IVF, stored on ice and measurements taken within 20 min of collection. We collected a standardised volume of 0.7–0.8 μL ejaculate per male. For Computer Assisted Sperm Analysis (CASA), 0.5 μL of semen‐buffer was placed on a four‐chamber 20‐μm slide (MicroTool B4 Slides; Cytonix) and activated with 1.5 μL system water at 28°C or 34°C. We activated sperm and measured sperm motility for each male at 28°C and 34°C using a heated microscope stage to keep the temperature constant during the 90 s time series. We used a brightfield microscope (UOPUB203i trinocular microscope; Proiser) at 10X magnification and a black and white video camera (782 M monochrome CCD progressive camera; Proiser). We used the CASA software ISASv1 (Proiser, R + D, S.L.) to measure sperm velocity with the following settings: frame rate 50 frames/s, 50 frames used, 5–50 μm^2^ particle area. We measured sperm parameters at 5 and 10 s after sperm activation, and every 10 s until 90 s, when 95% of sperm had stopped moving.

### 2.7 Sperm DNA Damage

We fixed semen‐buffer samples in 4% paraformaldehyde for 1 h at 4°C, then centrifuged the samples (350 g, 5 min) to replace paraformaldehyde with PBS and conserved them at 4°C until use. After three washes in PBS on a coverslip, sperm cells were permeabilised using 0.2% Triton X‐100 for 5 min on ice, and then washed again in PBS before incubation in a dark humidified chamber for 1 h at 37°C with the terminal deoxynucleotidyl transferase (TdT) reaction mix, which was prepared using the commercial kit Promega DeadEnd Fluorometric TUNEL System. We then repeated the PBS washes in darkness and assembled the microscope slides using Vectashield with DAPI from vectors laboratories as a mounting medium. We evaluated the DNA fragmentation level with a fluorescence microscope at 20X magnification, counting the number of sperm cells showing a green fluorescence over the healthy cells stained with DAPI. We also performed positive controls for all samples using a DNAse 1 treatment (Promega RQ1 RNase‐Free Dnase).

### 2.8 Statistical Analyses

We visualised and analysed all data in R v4.2.0 (R Core Team 2022), using Rstudio v2022.7.2.576 (RStudio Team 2022). We used the packages *dplyr* (Wickham, François, Henry & Müller, 2022) and *magrittr* (Bache and Wickham 2022) for data transformation, *ggplot2* (Wickham 2016), *ggbeeswarm* (Clarke, Sherrill‐Mix, and Dawson 2023), *RcolorBrewer* (Neuwirth 2022) and *shades* (Clayden 2019) for data visualisation, and *lme4* (Bates et al. 2014), *DHARMa* (Hartig and Lohse 2022), and *emmeans* (Lenth et al. 2024) for analysis.

We used linear (mixed) models for the Gaussian response variables sperm numbers, sperm motility and sperm velocity. Our fixed predictor variable for the models on sperm number was paternal treatment. Our fixed predictor variables for the models on sperm motility and velocity included paternal treatment, sperm activation temperature, time and their interactions. Additionally, we fitted a random intercept of a sample identifier nested into male to account for the repeated measures of sperm samples over time and the split ejaculate design. We used binomial generalised linear models (GLMs) and mixed GLMMs for proportion data (fertilisation, survival, normal development and DNA fragmentation), using *cbind* to combine successes and failures. To account for our split‐clutch design, we included random intercepts for individual clutches or males (Schielzeth and Forstmeier 2009). Random effects of tanks (unit of housing), blocks (experimental repeats over time), and sets or days (staggering of sets of two different treatment tanks, thus can be used as a tank identifier) were accounted for in models as random intercepts identified as ‘block/tank’ in Exp. 1 (tank was nested within block), ‘block/set’ in Exp. 2, ‘block/day’ in Exp. 3, and ‘block’ in Exp. 4 (only one large tank per treatment was used per block) and were removed if they did not explain a significant amount of the variance in the model. This was determined using an ANOVA comparison of models including and excluding random effects to determine if the model was significantly impacted (*p* < 0.05) by the removal of a variable. Overdispersion in binomial models was prevented by including observation‐level random intercepts (Harrison 2014). We obtained *p*‐values for fixed effects in binomial GLMMs from type‐III Wald tests implemented in the *car* package (Fox and Weisberg 2011), and in LMMs from *t*‐tests using Satterthwaite's approximation for denominator degrees of freedom implemented in *lmerTest* version 3.1–3 (Kuznetsova, Brockhoff, and Christensen 2017).

## 3 Results

### 3.1 Heatwaves Impair Sperm Quantity and Quality

**FIGURE 2:**
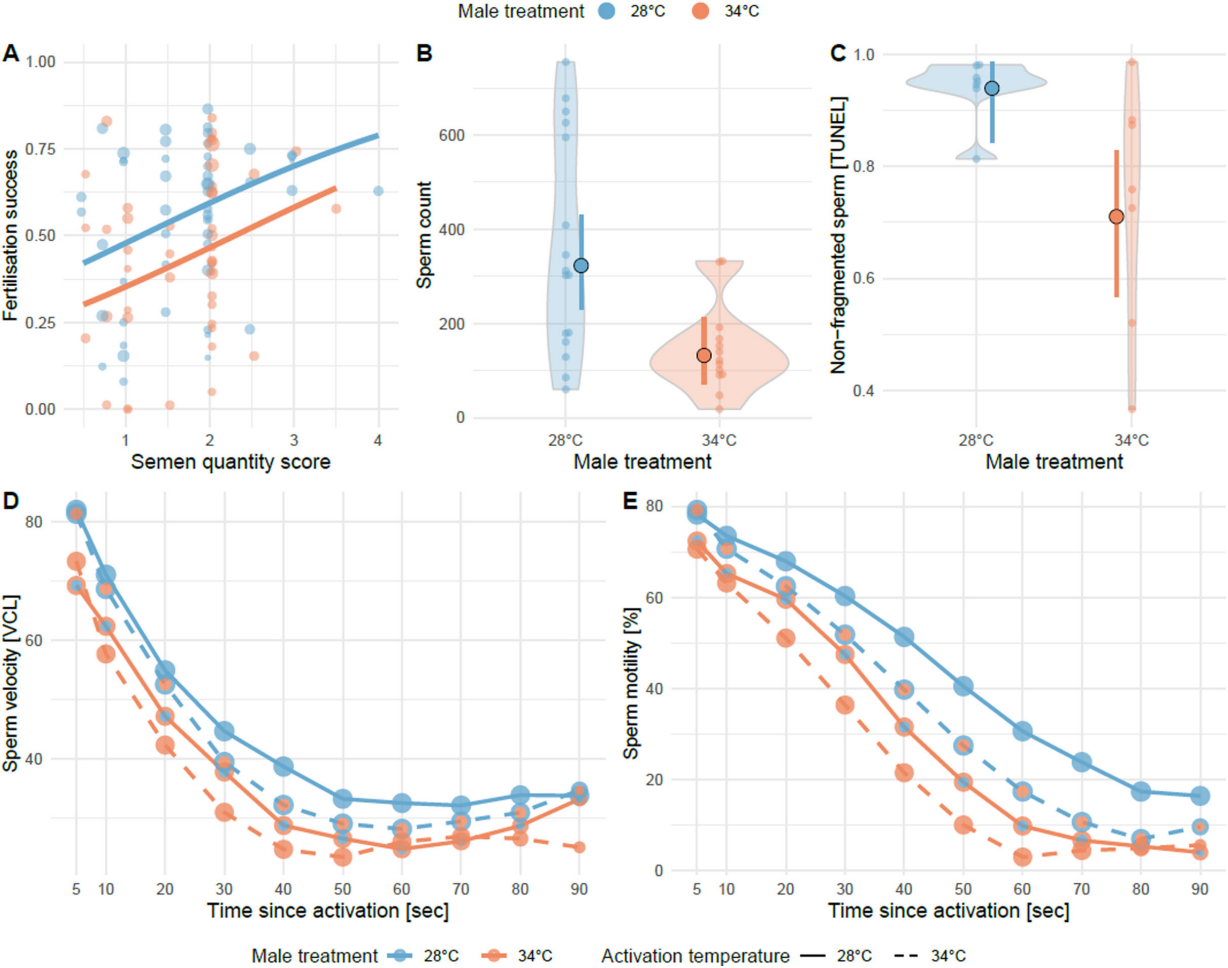
Effect of male heatwave exposure on ejaculate traits. Males were exposed to 34°C (red) or 28°C (blue) for 2 weeks. (A) Fertilisation success for 51 control clutches and 46 clutches sired by heatwave‐treated fathers was positively correlated with ejaculate volume collected for IVF but was consistently lower in males exposed to 34°C irrespective of ejaculate volume (lines and points represent model predictions and raw data, respectively, with symbol area proportional to clutch size). (B) Sperm density for ejaculates from 16 control males and 13 heatwave‐treated males, (C) Sperm DNA integrity in ejaculates from 7 control and 7 heatwave‐treated males (bold symbols and error bars in (B) and (C) depict model predictions for means and 95% confidence intervals, violin plots represent raw data), (D) sperm swimming velocity in ejaculates from 16 control and 13 heatwave‐treated males following activation of split ejaculates in control or heatwave temperatures and (E) sperm motility in ejaculates from 10 control and 10 heatwave‐treated males following activation of split ejaculates in control or heatwave temperatures (lines and circles in (D), (E) represent mean values with the number of samples proportional to circle size; solid lines indicate sperm activated at 28°C and dashed lines indicate sperm activated at 34°C).

We found no effect of heatwaves on the likelihood of spawning or retrieving a semen sample: 108 of 138 (78%) heatwave‐treated males spawned or contributed a semen sample, as did 107 of 136 (79%) control males (χ ^2^_1_ = 0, *p* = 1) in Exp. 3. Semen quantity was not lower for heatwave‐exposed males (*t*_1,95_ = −0.78, *p* = 0.436), and the effects of semen quantity on fertilisation success for control and heatwave‐exposed males were not significantly different (treatment x semen quantity interaction: χ ^2^_1_ = 0.01, *p* = 0.924; Figure 2A). The average sperm concentration in semen samples collected from heatwave‐exposed males was less than half of that from control males in Exp. 1 (LM square‐root‐transformed sperm count [CASA]: *b* [95% CI] = −6.48 [−10.62, −2.34], *t*_1,27_ = −3.2, *p* = 0.003; Figure 2B). Moreover, sperm from heatwave‐exposed males displayed a lower incidence of healthy sperm whose DNA was not fragmented in Exp. 1 (quasi‐binomial GLM on non‐fragmented sperm [TUNEL]: −1.85 [−3.48, −0.57], χ ^2^_1_ = 8.6, *p* = 0.003; Figure 2C).

Sperm motility was impeded by heatwave exposure during spermatogenesis, but this effect was dependent on time since activation (Gaussian LMER, male treatment × time since activation: −0.0007 [−0.001, −0.0002], χ ^2^_1_ = 6.24, *p* = 0.013). The same was true for the effect of heatwaves on mature sperm (Gaussian LMER, sperm treatment x time since activation: −0.001 [−0.002, −0.0008], χ ^2^_1_ = 6.41, *p* < 0.0001, Figure 2D, Table A2). Similarly, sperm velocity was reduced by heatwave exposure during spermatogenesis (Gaussian LMER: −8.85 [−15.27, −2.42], χ ^2^_1_ = 7.25, *p* = 0.007) and directly upon mature sperm, but this effect was again dependent on time since activation (Gaussian LMER, sperm treatment x time since activation: −0.09 [−0.13, −0.01], χ ^2^_1_ = 4.63, *p* = 0.031, Figure 2E, Table A2).

### 3.2 Heatwaves Lower Male Fertilisation Success

**FIGURE 3:**
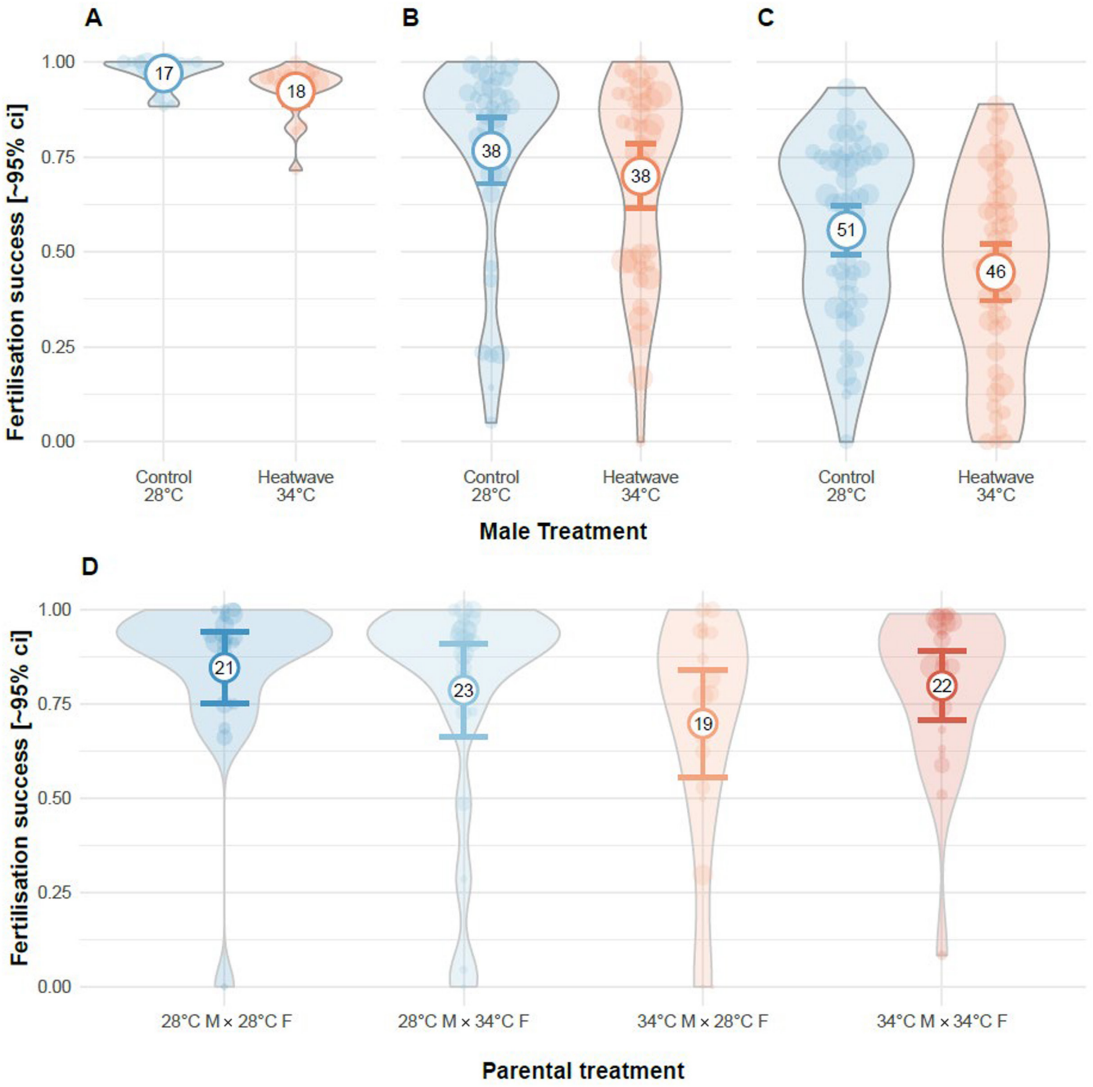
Effect of male heatwave exposure on fertilisation success. Males were exposed to 34°C (red) or 28°C (blue). (A) Exp. 1 (natural spawning), (B) Exp. 2 (natural spawning) and (C) Exp. 3 (IVF). The effect was significant in Exp. 1 and 3 but not in Exp. 2. In Exp. 4 (D), there was no effect of parental heatwaves on fertilisation success. Bold circles and error bars represent mean and 95% confidence intervals (sample size representing number of clutches obtained from breeding pairs indicated in numbers). Violin plots and open circles represent raw data and circle area is proportional to clutch size (dead eggs excluded).

In Exp. 1, fertilisation success was high throughout the experiment, however, heatwaves caused a small but significant reduction in male fertility (binomial GLMM: −1.04 [−1.61, −0.47], χ ^2^_1_ = 12.67, *p* < 0.001, Table A3). In Exp. 3, our visual score of semen quantity had a positive relationship with fertilisation success (binomial GLMM: 0.44 [0.09, 0.79], χ ^2^_1_ = 6.24, *p* = 0.013; Figure 2A), and fertilisation success was indeed lower for heatwave‐exposed males (binomial GLMM: −0.54 [−1.02, −0.06], χ ^2^_1_ = 4.84, *p* = 0.028; Figure 3B, Table A3), but sperm heatwave treatment had no effect (binomial GLMM: 0.10 [−0.38, 0.58], χ ^2^_1_ = 0.16, *p* = 0.69). In contrast, in Exp. 2 and 4, paternal and parental heatwave exposure, respectively, had no effect on fertilisation success (Exp 3: binomial GLMM: −0.57 [−1.35, 0.20], χ ^2^_1_ = 2.15, *p* = 0.143, Figure 3C; Exp 4.: Table A3 and Figure 3D). Additionally, in Exp. 4, maternal heatwave exposure had no effect on clutch production (binomial GLMM: −0.81 [−1.81, 0.13], χ ^2^_1_ = 2.83, *p* = 0.093) or clutch size (Gaussian LMM: 3.91 [−11.01, 20.65], χ ^2^_1_ = 0.27, *p* = 0.601).

### 3.3 Limited Evidence That Paternal Heatwave Effects Are Adaptive to Offspring Development

**FIGURE 4:**
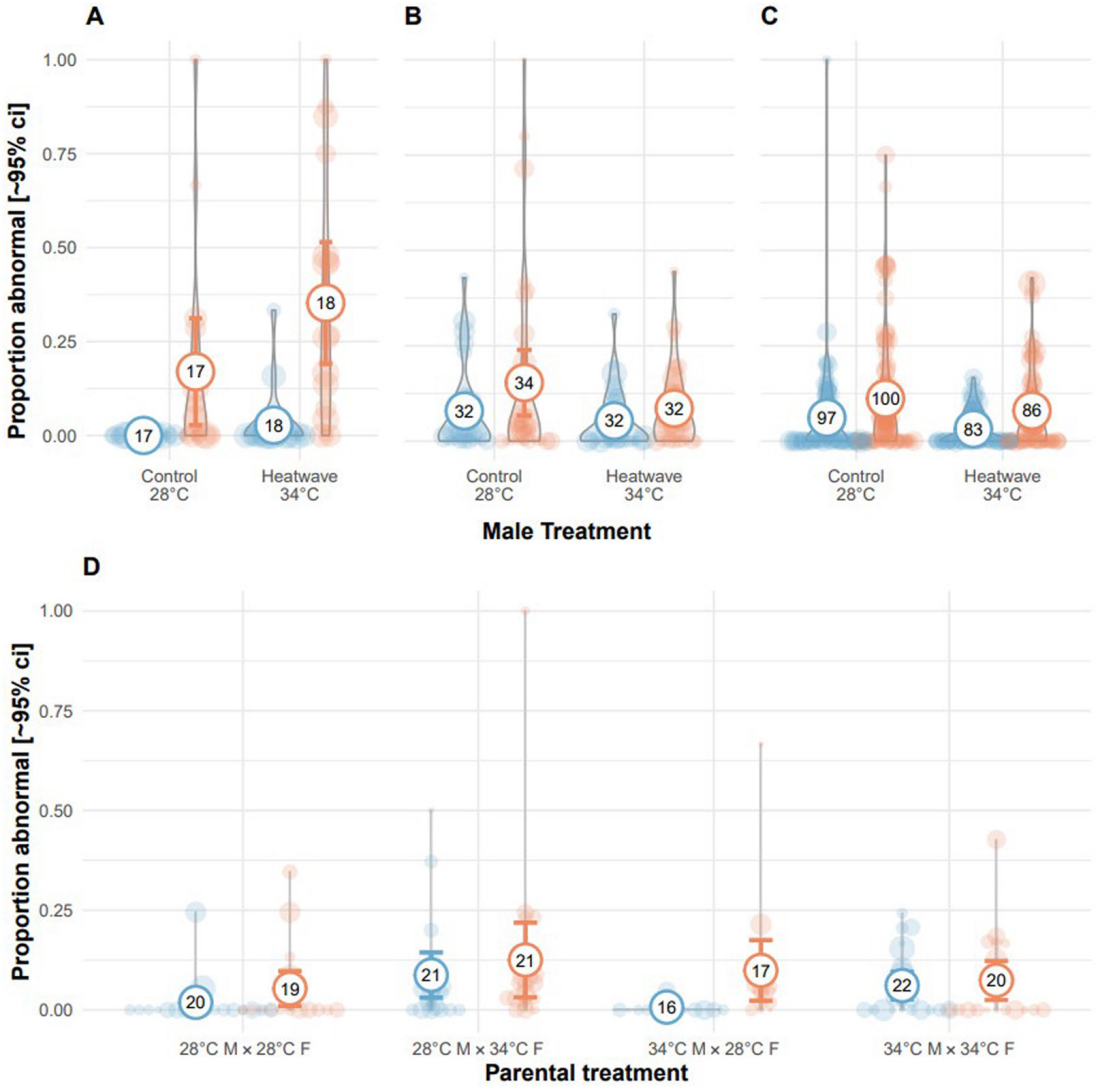
Effect of male heatwave exposure on normal offspring development. Males were exposed to 34°C or 28°C. (A) In Exp. 1, the ratio of abnormal embryos at 120 hpf was lower in clutches sired by males exposed to 34°C regardless of offspring treatment (red: 34°C; blue: 28°C). (B) In Exp. 2, the ratio of abnormal embryos 24 hpf was not affected by paternal treatment, and (C) in Exp. 3 the ratio of abnormal embryos 24 hpf was reduced in clutches reared at 34°C when sired by males exposed to 34°C. In Exp. 4 (D), rates of abnormal offspring 24 hpf in clutches produced by pairs with a female exposed to a two‐week heatwave, in control offspring was higher than that of control females. Bold circles and error bars represent mean and 95% confidence intervals (sample size representing the number of clutches (after splitting) that have survived to 24 hpf indicated in numbers). Violin plots and open circles represent raw data, with circle area proportional to clutch size (dead eggs excluded).

In Exp. 1, as observed 120 h post‐fertilisation, clutches from either treatment or control temperatures fertilised by heatwave‐exposed males had higher rates of developmental abnormalities (binomial GLMM: 1.80 [0.13, 3.47], χ ^2^_1_ = 4.45, *p* = 0.035; Figure 4A). However, in Exp. 3, heatwave‐exposed males produced offspring with lower rates of abnormalities at 24 hpf compared to control males (binomial GLMM: −0.41 [−0.79, −0.03], χ ^2^_1_ = 4.43, *p* = 0.035; Figure 4B), and sperm treatment had no effect on offspring development (binomial GLMM: −0.061 [−0.33, 0.21], χ ^2^_1_ = 0.200, *p* = 0.655, Table A4), regardless of embryo treatment. In Exp. 2, there was no effect of male heatwave exposure on normal development (binomial GLMM: −0.46 [−1.04, 0.12], χ ^2^_1_ = 2.41, *p* = 0.120), and only embryo heatwave exposure increased the presence of abnormalities in the offspring (binomial GLMM: 0.63 [0.03, 1.24], χ ^2^_1_ = 4.23, *p* = 0.040; Figure 4C, Table A4). Exp. 2, 3 and 4 recorded abnormalities at 24 hpf, compared to observations performed much later in Exp. 1. In Exp. 4, clutches produced from heatwave‐exposed females had significantly more offspring with developmental abnormalities when clutches were reared in control temperatures (binomial GLMM—female x embryo treatment: −1.73 [−2.95, −0.52], χ ^2^_1_ = 7.85, *p* = 0.005; Figure 4D, Table A4).

**FIGURE 5:**
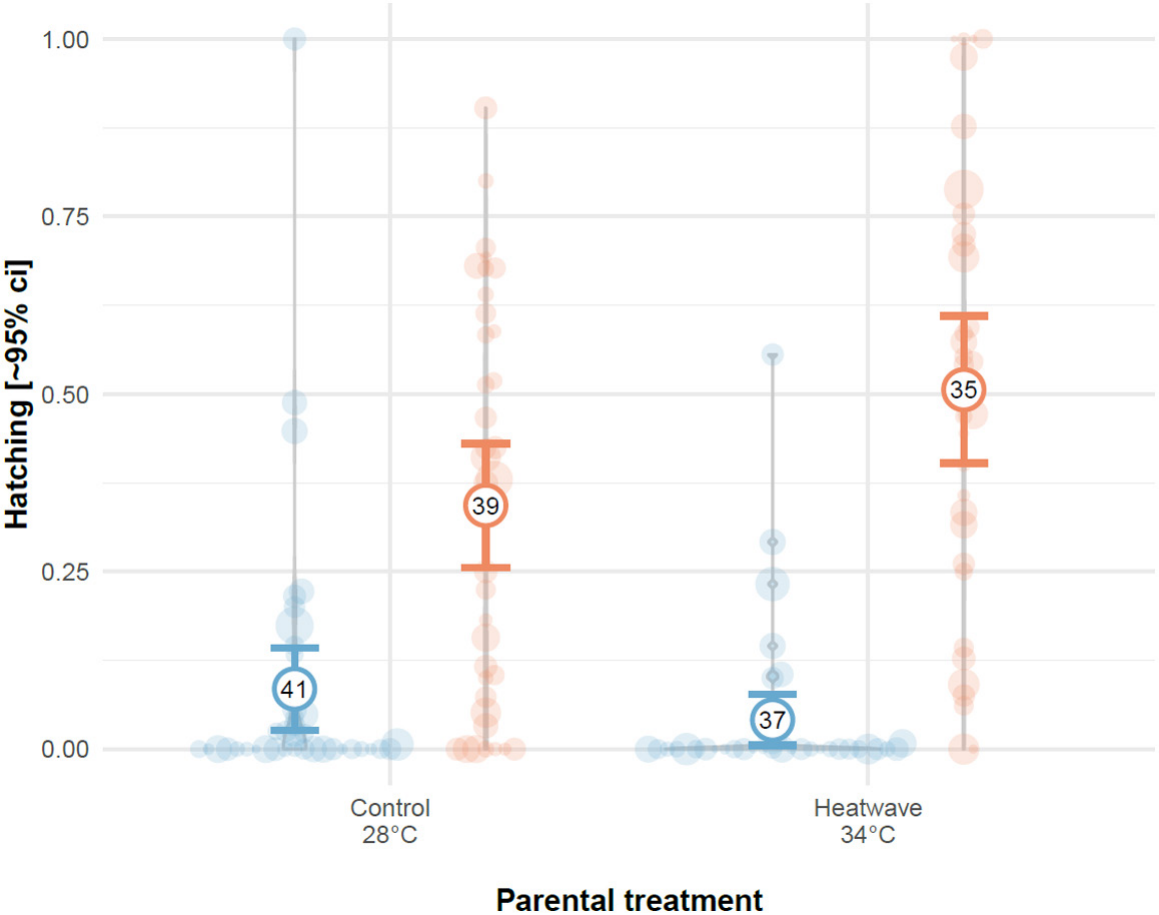
Effect of male heatwave exposure on offspring hatching rates (percentage of offspring hatched per clutch) at 48 hpf in Exp. 4. Hatching rates were faster in clutches produced by pairs with males exposed to a two‐week heatwave, when offspring experienced heatwave temperatures (34°C: Red, 28°C: Blue). Bold circles and error bars represent mean and approximate 95% confidence intervals (sample size representing number of surviving clutches at 48 hpf indicated in numbers). Violin plots and open circles represent raw data, with circle area proportional to clutch size (dead eggs excluded).

Furthermore, paternal heatwave exposure may induce earlier hatching rates in their clutches. In Exp. 4, heatwave‐exposed clutches produced by heatwave‐exposed males had the highest rates of hatching at 48 hpf (binomial GLMM—male x embryo treatment: 2.06 [0.72, 3.39], χ ^2^_1_ = 9.06, *p* = 0.003, Figure 5, Table A5).

### 3.4 No Evidence That Paternal Heatwaves Affect Offspring Survival

**FIGURE A1:**
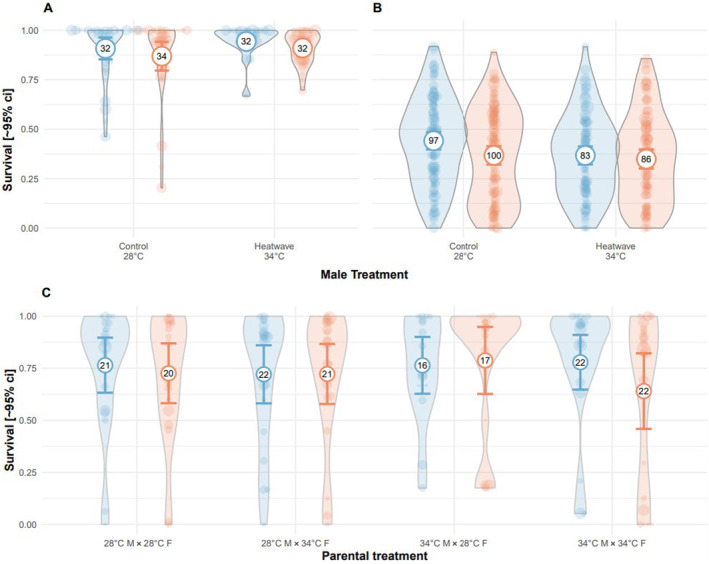
Effect of male heatwave exposure on offspring survival. Males were exposed to 34°C or 28°C. Survival rates of clutches were not affected by paternal treatment regardless of offspring treatment (red: 34°C; blue: 28°C) in Exp. 2 (A) and 3 (B). In Exp. 4 (C), neither maternal nor paternal treatment affected clutch survival rates across any parental treatment combinations and offspring treatments. Bold circles and error bars represent mean and 95% confidence intervals (sample size representing the number of clutches (after splitting) that have survived to 24 hpf indicated by numbers). Violin plots and open circles represent raw data, with circle area proportional to clutch size.

Paternal heatwave exposure had no effect on embryo survival in Exp. 2 and 3 (binomial GLMMs: Exp 2: −0.02 [−0.69, 0.66], χ ^2^_1_ = 0.002, *p* = 0.960; Exp 3: −0.30 [−0.92, 0.32], χ ^2^_1_ = 0.88, *p* = 0.349, Figure A1A,B, Table A6), and neither maternal (binomial GLMM: −0.18 [−1.18, 0.82], χ ^2^_1_ = 0.49, *p* = 0.724) nor paternal (binomial GLMM: 0.36 [−0.64, 1.35], χ ^2^_1_ = 0.12, *p* = 0.482) heatwave exposure impacted offspring survival in Exp. 4 (Figure A1C, Table A6).

See Table 1 for an overview of all results.

**TABLE 1:** Summary of results across all four experiments indicating the paternal effects of increased temperature on male fertility and offspring phenotypes. The ‘–’ indicates significantly negative effects and the ‘+’ indicates significantly positive effects, whereas ‘n.s.’ indicates no significant effects in either direction.

Embryo treatment	No. of fertilised eggs	No. of dead embryos (24 hpf)		No. of abnormal embryos (120 hpf (Exp. 1) or 24 hpf)		No. of hatched larvae (48 hpf)	
		28°C	34°C	28°C	34°C	28°C	34°C
Exp. 1	−	n.s.	n.s.	+	+	NA	NA
Exp. 2	n.s.	n.s.	n.s.	n.s.	n.s.	NA	NA
Exp. 3	−	n.s.	n.s.	−	−	NA	NA
Exp. 4 ‐paternal	n.s.	n.s.	n.s.	n.s.	n.s.	n.s.	+
Exp. 4 ‐maternal	n.s.	n.s.	n.s.	+	n.s.		

## 4 Discussion

Our results indicate that an increase in temperature in the form of heatwaves over a relatively short period of time has negative effects on male reproduction and fertility in the form of fewer gametes and lower fertilisation success rates in individuals exposed to heatwaves (Table A2). However, the evidence for inter‐generational effects of such heatwaves on early offspring development is limited and inconclusive. We found indications of negative paternal and maternal effects on offspring survival but interestingly, offspring from heatwave‐exposed males hatched slightly earlier which could give them a selective advantage. In contrast, maternal effects of female exposure to heatwaves are primarily detrimental and had only negative effects on early development in control offspring. Overall, we found limited evidence for an adaptive role of inter‐generational effects in our study. We discuss our results in detail here below and compare them to findings in other ectotherms.

### 4.1 Temperature Effects on Reproductive Fitness

Following heatwave exposure, male zebrafish exhibited reduced sperm concentration and quality, which are likely the factors that contribute to the reduced fertility observed in Exp. 1 and 3. However, heatwaves during sperm activation also reduced sperm motility and velocity, indicating that both indirect exposure to heatwaves during spermatogenesis in the male and direct exposure during activation just before fertilisation may have detrimental effects on fertility in externally fertilising organisms. These results are somewhat inconsistent with findings in Brook charr *Salvelinus fontinalis*, which suggested that due to epigenetic differences in offspring following paternal exposure to thermal stress during maturation, spermatogenesis rather than mature sperm physiology is vulnerable to thermal stress (Venney et al. 2022). While zebrafish can be found at high temperatures in the wild, as high as 34°C (Morgan et al. 2019), their populations may not actually be sustainable at these temperatures. Due to the heightened sensitivity of male and female reproductive systems, population maintenance and growth may be compromised at temperatures lower than their upper CTL. In addition, thermal tolerance in the laboratory might be an overestimate of wild tolerance, due to the otherwise benign conditions allowing the fish to compensate (Morgan et al. 2019). Our findings therefore support the idea that TFL is a key factor to consider when assessing the ability of a species to cope with short‐term drastic increases in temperature.

### 4.2 Effects of Temperature on Offspring Development

In Exp. 3, we observed lower rates of 24 hpf developmental abnormalities in offspring produced by thermally stressed fathers, compared to those produced by control fathers. In contrast to these results, in Exp. 1 thermally stressed fathers appear to have produced more abnormal offspring at 120 hpf than control fathers regardless of offspring thermal environment. However, detrimental maternal thermal stress effects in Exp. 4 may have outweighed any effects of paternal thermal stress. This may indicate that the effect of maternal thermal stress on offspring may mask that of fathers, especially considering the high amount of variation we observed following paternal thermal stress. Since spawning females and males will likely experience the same environmental conditions leading up to their reproductive period, paternal effects may not be enough to benefit subsequent generations, and that climate change may pose problems for offspring viability regardless.

Our results from Exp. 1 are more in line with other experiments that have found detrimental effects in offspring following paternal thermal stress, as was found in *G. bimaculatus*, where offspring experienced lower hatching success and survival (Gasparini et al. 2018), and *T. castaneum* whose offspring had shorter lifespan and reduced reproduction (Sales et al. 2018). However, it is unclear what role selective disappearance may have played in controlling offspring in these experiments. These organisms are also internal fertilisers, which may have gametes less adapted to potential environmental stressors. In *G. caespitosa*, an external fertiliser, did exhibit reduced offspring survival following acute paternal thermal stress, as in Exp. 1, but allowing for acclimation mitigated this (Guillaume, Monro, and Marshall 2016). Our results from Exp. 3 showed the opposite trend, more closely resembling other studies in fish where thermally stressed parents (and grandparents; Munday, Donelson, and Domingos 2017) produced offspring that performed better in similarly thermally stressful environments (Munday, Donelson, and Domingos 2017; Salinas and Munch 2012). Although our results from Exp. 4 indicate a stronger, detrimental effect of maternal thermal stress, unlike those experiments.

Due to the unique ecologies and physiologies of the animals described here, it is difficult to ascertain whether differing results between studies are due to these disparities between study systems or differences in methods. Some organisms were allowed to acclimate to their temperature environments (Guillaume, Monro, and Marshall 2016; Munday, Donelson, and Domingos 2017; Salinas and Munch 2012) whereas our experiments used acute thermal stress. The wide variation in the effects of thermal stress we see across studies is likely the result of not just differing experimental design, but physiology, ecology and even individual variation as shown in our own experiments.

The evidence of an adaptive effect of parental exposure to heatwaves in the next generation in our experiments is limited, as our results were highly variable. However, further indication that paternal heatwave exposure in males may convey adaptive advantages was observed in Exp. 4, where thermally stressed offspring hatched earlier when sired by crosses with heatwave‐exposed males. Early hatching can convey an advantage in externally fertilising fish as suggested by findings in the Atlantic salmon *Salmo salar* where early hatched larvae have better access to food resources (Brännäs 1995). In salmonids more generally, juveniles, and immobile embryos in particular, tend to be the most vulnerable to environmental challenges such as predation and food availability (Elliott 1986; Henderson and Letcher 2003), a pattern which may well apply to other fish species. In a thermally stressful environment, this could be particularly important as climatic events often coincide with changes in resource availability (IPCC 2023 *)*. However, life history theory posits that there are trade‐offs between early and late‐life fitness (Kirkwood 1977) and rapid growth in early life could result in detrimental effects later (Lee, Monaghan, and Metcalfe 2013).

Males may be more capable of responding in a favourable way for their offspring in the face of environmental challenges due to their ongoing cycles of spermatogenesis. Females, on the other hand, may be more likely to accumulate damage in their oocytes due to external stressors, which they are unable to regenerate. In teleosts, final oocyte maturation leading up to spawning is especially sensitive to elevated temperatures, resulting in impaired reproduction and oocyte defects (Alix, Kjesbu, and Anderson 2020). This may explain why we observed more detrimental effects under maternal exposure to heatwaves compared to paternal exposure. Furthermore, the effects of thermal stress we observe on sperm and male fertility may indicate that fewer damaged sperm are able to reach the fertilisation process, thus reducing the potential for detrimental effects of paternal thermal stress on offspring. While ejaculates and sperm carry heritable non‐genetic factors that can affect the following generation(s), there is currently limited evidence that these effects are adaptive (Godden and Immler 2023; Immler 2018; Valdivieso et al. 2020). Furthermore, matching environments between parents and offspring may be key to adaptive potential, as Jensen and colleagues found that offspring of marine tubeworm *Hydroides diramphus* survived better in salinity conditions that their parents experienced but performed poorly in salinity conditions that their parents did not experience (Jensen, Allen, and Marshall, 2014). Under rapid, transient environmental changes adaptive effects may then be limited in both time and space. However, such effects could become more important under long‐term exposure to heatwaves and a more consistent change in temperature (Silva, Otto, and Immler 2021). Whether such responses are swift enough to warrant the survival of a species facing severe temperature changes over a short period of time is still in question.

## 5 Conclusions

Overall, we found reductions in male fertility due to acute thermal stress and limited evidence for inter‐generational adaptation via paternal non‐genetic inheritance, but maternal inheritance may be more important. Although some marine fish species are expected to acclimate to increasing water temperatures (Donelson et al. 2012), other species may be less likely to cope. In fact, zebrafish appear to have an upper temperature limit and a lower capacity for adaptation to higher temperatures than to low temperatures (Morgan et al. 2020). Such high temperatures appear to impose limits on male and female fertility, which could influence population stability in nature under current climate change projections (IPCC 2023). Our study supports the idea that we need to include TFLs in addition to CTLs to accurately assess the impacts of climate change on wild populations.

## Author Contributions

**Sara D. Irish:** conceptualization (equal), data curation (equal), formal analysis (lead), investigation (equal), project administration (equal), visualization (equal), writing – original draft (lead), writing – review and editing (lead). **Andreas Sutter:** conceptualization (equal), data curation (equal), formal analysis (supporting), investigation (equal), methodology (equal), project administration (equal), visualization (equal), writing – original draft (supporting), writing – review and editing (supporting). **Livia Pinzoni:** conceptualization (equal), data curation (equal), investigation (equal), methodology (equal), project administration (equal), writing – review and editing (supporting). **Mabel C. Sydney:** conceptualization (equal), data curation (equal), investigation (equal), methodology (equal), project administration (equal), writing – review and editing (supporting). **Laura Travers:** investigation (equal), project administration (equal), writing – original draft (supporting), writing – review and editing (supporting). **David Murray:** methodology (equal), project administration (equal), writing – review and editing (supporting). **Jean‐Charles de Coriolis:** project administration (equal), writing – review and editing (supporting). **Simone Immler:** conceptualization (equal), funding acquisition (lead), investigation (equal), resources (lead), supervision (lead), writing – original draft (supporting), writing – review and editing (supporting).

## Ethics Statement

The experiments in this study strictly followed UK Home Office guidelines and were performed under Project Licence No. P0C37E90*1*.

## Conflicts of Interest

The authors declare no conflicts of interest.

## Data Availability

The data collected and code used for analysis that support our findings are available in Dryad Data Repository and can be accessed at https://doi.org/10.5061/dryad.hx3ffbgn0.

## Appendix A

**TABLE A1:** Numbers of fish, tanks, blocks and treatments for each of the four experiments are presented as used for statistical analyses. If more than one number is presented this reflects the number of fish/tanks per treatment (Control, Heat). Each of the experiments described was performed by placing fish in each treatment into several tanks and in more than one block. Tank and block were taken into account as random intercepts (see Statistical Analyses below for details) where appropriate (tank and block may overlap).

Experiment	No. of fish	No. of tanks	No. of blocks	No. treatments
Exp. 1	18, 17	6,6	3	2
Exp. 2	38, 38	10, 10	5	2
Exp. 3	102, 92	10,10	5	2
Exp. 4	Males: 41, 38 Females: 42, 46	6, 6 6, 6	6	2 2

**TABLE A2:** Linear mixed effects model (*lmer* in R) on sperm motility and sperm velocity following paternal heatwaves and heatwave treatments during sperm activation. Data obtained through CASA from Exp. 1.

Effect	Sperm motility				Sperm velocity			
	Est.	*χ* ^*2*^_*1*_	Cis	*p*	Est.	*χ* ^*2*^_*1*_	Cis	*p*
Time	−0.009	1379.42	−0.009, −0.008	**< 0.0001**	−0.56	648.74	−0.60, −0.20	**< 0.0001**
Male treatment	−0.11	3.50	−0.23, −0.004	0.061	−8.85	7.25	−15.27, −2.42	**0.007**
Sperm treatment	−0.03	2.18	−0.08, −0.01	0.139	−0.76	0.23	−3.86, 2.33	0.629
Male treatment × time	−0.001	6.24	−0.001, −0.0002	**0.013**	—	—	—	—
Sperm treatment × time	−0.001	21.35	−0.002, −0.001	**< 0.0001**	−0.07	4.63	−0.13, −0.01	**0.032**
	**Random effects variance:** Sample: male: 0.008, SD = 0.091 Male: 0.022, SD = 0.147				**Random effects variance:** Sample: male: 6.381, SD = 2.526 Male: 70.762, SD = 8.412			

*Note:* *p*‐values < 0.05 are indicated in bold.

**TABLE A3:** Binomial GLMM (*glmer* in R) for effects of parental heatwave treatments (paternal heatwave (M HW), maternal heatwave (F HW) and the interaction between them (M × F HW) on fertilisation rate measured 2‐h after eggs are laid, with response variable *cbind* (fertilised eggs, unfertilised eggs). No statistics presented means that this trait was not assessed in that experiment.

Effect	Exp. 1				Exp. 2				Exp. 3				Exp. 4			
	Est.	*χ* ^*2*^_*1*_	Cis	*p*	Est.	*χ* ^*2*^_*1*_	Cis	*p*	Est.	*χ* ^*2*^_*1*_	Cis	*p*	Est.	*χ* ^*2*^_*1*_	Cis	*p*
Sperm quantity	—	—	—	—	—	—	—	—	0.44	6.23	0.09, 0.79	**0.01**	—	—	—	—
SPERM treatment	—	—	—	—	—	—	—	—	0.10	0.16	−0.38, 0.58	0.69	—	—	—	—
Male treatment	−1.04	12.67	−1.61, −0.47	**< 0.001**	−0.61	2.89	−1.32, 0.10	0.09	−0.54	4.84	−1.02, −0.06	**0.03**	−0.68	2.54	−1.55, 0.17	0.11
Female treatment	—	—	—	—	—	—	—	—	—	—	—	—	0.08	0.04	−0.76, 0.91	0.85
Male × female	—	—	—	—	—	—	—	—	—	—	—	—	0.64	0.60	−1.03, 2.31	0.44
Random effects variance:					Male_ID: 2.264, SD = 1.505								Ind_ID: 0.860, SD = 0.928 Clutch.ID: 3.188, SD = 1.786			

*Note:* *p*‐values < 0.05 are indicated in bold.

**TABLE A4:** Binomial GLMM (*glmer* in R) for effects of parental and offspring heatwave treatments on offspring abnormality rate per clutch measured at 120‐h post‐fertilisation (Exp. 1) and 24‐h post‐fertilisation (Exp. 2, 3, & 4), with response variable *cbind* (abnormal eggs, normal eggs). Post hoc analysis using emmeans() shows where significant interactions were present. No statistics presented means that this trait was not assessed in that experiment.

Effect	Exp. 1				Exp. 2				Exp. 3				Exp. 4			
	Est.	*χ* ^*2*^_*1*_	Cis	*p*	Est.	*χ* ^*2*^_*1*_	Cis	*p*	Est.	*χ* ^*2*^_*1*_	Cis	*p*	Est.	*χ* ^*2*^_*1*_	Cis	*p*
Male treatment	1.80	4.45	0.13, 3.47	**0.035**	−0.46	2.41	−1.04, 0.12	0.12	−0.41	4.43	−0.79, −0.03	**0.035**	0.97	2.00	−0.38, 2.32	0.16
Female treatment	—	—	—	—	—	—	—	—	—	—	—	—	2.90	15.09	1.44, 4.36	**< 0.001**
Sperm treatment	—	—	—	—	—	—	—	—	−0.06	0.20	−0.33, 0.21	0.65	—	—	—	—
Offspring treatment	4.27	59.95	3.19, 5.35	**< 0.001**	0.65	6.10	0.13, 1.17	**0.014**	0.97	48.72	0.70, 1.25	**< 0.001**	2.17	18.89	1.19, 3.15	**< 0.001**
Male × female	—	—	—	—									−1.74	3.68	−3.52, 0.04	0.06
Female × Offspring	—	—	—	—	—	—	—	—	—	—	—	—	−1.73	7.85	−2.95, −0.52	**< 0.01**
Random effects variance:	Male_ID: 3.444, SD = 1.856				Ind_ID: 1.985, SD = 1.409				Ind_ID: 0.570, SD = 0.755 Male_ID: 0.398, SD = 0.631 Block: 0.123, SD = 0.351				Ind_ID: 1.652, SD = 1.285 Clutch.ID: 2.019, SD = 1.421			
									Female × offspring post hoc analysis				*Est*.	*Z*	*SE*	*p*
									Offspring 28°C, F 28°C—34°C				−2.03	−3.46	0.59	**< 0.001**
									Offspring 34°C, F 28°C—34°C				−0.29	−057	0.51	0.568

*Note:* *p*‐values < 0.05 are indicated in bold.

**TABLE A5:** Binomial GLMM (*glmer* in R) for effects of parental and offspring heatwave treatments on offspring early hatching rates measured at 48 h post‐fertilisation in Exp. 4, with response variable *cbind* (Hatched eggs, unhatched eggs). Post hoc analysis using *emmeans*() shows where significant interactions were present.

Effect		Exp. 4			
		Est.	*χ* ^*2*^_*1*_	Cis	*p*
Male treatment		0.04	0.01	−0.77, 0.85	0.88
Female treatment		0.55	1.88	−0.23, 1.35	0.08
Offspring treatment		3.85	107.31	3.12, 4.57	**< 0.0001**
Male **×** Offspring		2.06	8.48	0.68, 3.45	**0.004**
Random effects variance		Ind_ID: 2.997, SD = 1.731		Clutch_ID: 0.886, SD = 0.941	
	**Male × offspring post hoc analysis**	*Est*.	*Z*	*SE*	*p*
	Offspring 28°C, M 28°C—34°C	1.09	1.92	0.57	0.055
	Offspring 34°C, M 28°C—34°C	−0.97	−2.06	0.47	**0.039**

*Note:* *p*‐values < 0.05 are indicated in bold.

**TABLE A6:** Binomial GLMM (*glmer* in R) for effects of parental and offspring heatwaves treatments on offspring mortality rate per clutch measured at 24‐h post‐fertilisation, with response variable *cbind* (dead eggs, alive eggs). No statistics presented means that this trait was not assessed in that experiment.

	Exp. 2				Exp. 3				Exp. 4			
Effect	Est.	*χ* ^*2*^_*1*_	Cis	*p*	Est.	*χ* ^*2*^_*1*_	Cis	*p*	Est.	*χ* ^*2*^_*1*_	Cis	*p*
Male treatment	−0.30	0.88	−0.92, 0.32	0.35	−0.02	0.002	−0.69, 0.66	0.96	0.36	0.12	−0.64, 1.35	0.48
Female treatment	—	—	—	—	—	—	—	—	−0.18	0.49	−1.35, 0.51	0.72
Offspring treatment	0.67	5.64	0.12, 1.21	**0.02**	0.17	5.85	0.03, 0.30	**0.02**	−0.40	1.27	−1.08, 0.29	0.26
Random effects variance	Ind_ID: 2.407, SD = 1.551				Ind_ID: 0.334, SD = 0.578 Male_ID: 2.545, SD = 1.595 Block: 0.391, SD = 0.625				Ind_ID: 2.169, SD = 1.473 Clutch_ID: 3.219, SD = 1.794			

*Note:* *p*‐values < 0.05 are indicated in bold.
